# Relationship Between Oral Function and Social Participation Among Community-Dwelling Older Adults: An Observational Cross-Sectional Study

**DOI:** 10.3390/healthcare13182271

**Published:** 2025-09-11

**Authors:** Mayu Takeda, Yuhei Matsuda, Takafumi Abe, Kazumichi Tominaga, Hisaaki Saito, Jun Shimizu, Norikuni Maeda, Ryouji Matsuura, Yukio Inoue, Yuichi Ando, Shozo Yano, Minoru Isomura, Takahiro Kanno

**Affiliations:** 1 Department of Oral and Maxillofacial Surgery, Shimane University Faculty of Medicine, Izumo 693-8501, Shimane, Japan; mtakeda@med.shimane-u.ac.jp (M.T.); yuhei@med.shimane-u.ac.jp (Y.M.); 2 Center for Community-Based Healthcare Research and Education (CoHRE), Head Office for Research and Academic Information, Shimane University, Izumo 693-8501, Shimane, Japan; t-abe@med.shimane-u.ac.jp (T.A.); shika-t@ohtv.ne.jp (K.T.); kotobuki@iwami.or.jp (H.S.); syano@med.shimane-u.ac.jp (S.Y.);; 3 Shimane Dental Association, Matsue 690-0884, Shimane, Japanmatsuba@krf.biglobe.ne.jp (R.M.);; 4 National Institute of Public Health, Wako 351-0197, Saitama, Japan; ando.y.aa@niph.go.jp; 5 Department of Laboratory Medicine, Shimane University Faculty of Medicine, Izumo 693-8501, Shimane, Japan; 6 Faculty of Human Sciences, Shimane University, Matsue 690-8504, Shimane, Japan

**Keywords:** oral function, oral frailty, oral hypofunction, social participation, older adults

## Abstract

**Background and Objectives**: Pre-frailty is characterized by a lack of social interaction, mental instability, and decreased interest in health behaviors and oral health. Thus, this study aimed to explore the relationship between oral function and social participation among community-dwelling older adults. **Methods**: The study participants were community-dwelling older adults who underwent dental and oral health examinations and health checkups conducted by the Shimane Extended Union of the Medical Care System for Latter-Stage Elderly People between April 2020 and March 2022. General background data, oral health status, and social participation data were collected. Logistic regression analysis was performed as the primary analysis, with social participation as the objective variable. **Results**: The participants included 4196 cases, excluding 513 cases with missing data. Logistic regression analysis of the presence of going out at least once a week demonstrated significant correlations in age, lower leg circumference, masticatory function, and oral hygiene status (*p* < 0.05). Significant correlations were found in sex, lower leg circumference, masticatory function, swallowing function, and oral hygiene status for regular meetings with family or friends (*p* < 0.05). **Conclusions**: There may be an association between social participation and a decline in oral function and hygiene status among community-dwelling older adults.

## 1. Introduction

The older population in Japan has been increasing annually and was reported to be 36.23 million in 2023 [1]. Japan’s aging rate of 29.1% was the highest ever recorded and among the highest in the world [2]. The aging population is likely to continue to increase with the growing older population and the declining younger generation. In comparison with other countries, Japan’s aging rate is remarkably high. For example, in 2024, 20.1% of the population in South Korea, 19.4% in Spain, and 21.8% in Portugal were predicted to be aged 65 years or older [3,4]. In China, this proportion was 14.9% in 2022. These figures highlight the exceptionally high proportion of the aged population in Japan [5]. Aging decreases physical function and increases the incidence of noncommunicable diseases. Owing to the increase in the older population in Japan, the number of people requiring support and care is also increasing annually. The condition that precedes the need for nursing care is called frailty, and frail prevention is considered important from the perspective of preventing nursing care [6]. Frailty is a state in which the ability to maintain health becomes fragile owing to age-related changes in physical function and loss of reserve capacity [7]. Frailty is defined as a reversible condition that can be reversed to a healthy state through medical intervention [8]. Frailty requires multifaceted medical and health interventions due to the interplay among physical, mental, psychological, and social factors. Thus, countermeasures for the older adults in Japan have focused on nursing care prevention.

In Japan, the 8020 campaign was employed in 1989 through comprehensive medical cooperation in the community to encourage people to keep at least 20 of their own teeth at the age of 80 [9]. As a result of the 8020 campaigns, the oral health status of the older adults in Japan improved over the next 20 years and was eventually included in one of the health promotion laws [10]. In 2016, the introduction of the concepts of oral frailty and oral hypofunction marked a paradigm shift—from focusing solely on preserving remaining teeth to emphasizing the maintenance of overall oral function. Oral frailty describes the progressive decline of the teeth, tongue, and perioral muscles, which can ultimately result in physical impairments such as malnutrition and dysphagia. Although a decline in oral function is often viewed as a simple aging phenomenon, if left untreated, it is a risk factor that can lead to poor nutritional intake, social isolation, and even the need for nursing care and thus requires early intervention. Oral hypofunction, the next stage of oral frailty, is defined as a condition in which there is no minor loss of function, but rather a combined loss of oral function on oral examination. If left untreated, this oral hypofunction leads to oral dysfunction, such as mastication and swallowing disorders, leading to the progression of malnutrition and sarcopenia and the need for nursing care. In Japan, oral hypofunction can be diagnosed as a dental disease and was included in the National Health Insurance guidelines in April 2018. A 2019 report highlighted that oral health had long been excluded from broader medical policy, leading to structural issues including an overemphasis on treatment, reliance on market forces, and the influence of the sugar industry. This recognition has contributed to raising awareness in Japan regarding the importance of maintaining dental health and overall oral function [11]. Oral hypofunction is diagnosed by seven oral function measurements, and three or more items of decline are applicable; the seven oral function assessments are as follows: 1. poor oral hygiene, 2. oral dryness, 3. poor occlusal force, 4. poor tongue–lip motor function, 5. low tongue pressure, 6. poor masticatory function, and 7. poor swallowing function [2].

In the progression from health to frailty and physical dysfunction, the step prior to frailty is referred to as pre-frail [12]. Pre-frail is characterized by a lack of social interaction and mental instability, which leads to a decrease in spontaneity of health behaviors and, concurrently, a decline in interest in oral health [13]. Decreased interest in oral health can lead to the neglect of minor problems, such as dental caries, periodontitis, and xerostomia, increasing the risk of dental disease progression. This small change initiates the transition to oral frailty. This subjective awareness of oral health is called oral health literacy. Oral health literacy is a concept that defines knowledge regarding oral health and the ability to understand and make judgments when applying this knowledge to actual healthcare [14]. This refers not only to knowledge about dental caries and periodontal disease but also to the ability to correctly understand information and manage one’s own behavior based on this information to maintain overall oral health. People with low oral health literacy are reported to have poorer periodontal health and are more likely to develop periodontal disease [15]. Furthermore, people with low oral literacy are unable to take appropriate oral hygiene actions [16]. In addition, it has been suggested that health literacy and social behaviors are related. Low health literacy is significantly associated with fewer opportunities and frequency of receiving social support, with particularly strong effects among socially isolated older adults [17]. Additionally, older adults who ate meals alone had a significantly increased risk of worsening frailty [18]. Therefore, when the concepts of oral frailty and oral hypofunction were defined in 2016, it seemed reasonable to encompass the association between oral and social behavioral impairments in the conceptual framework [19]. However, to date, there has been no clear evidence demonstrating an association between this minor decline in oral function and social behavior, and a highly sensitive, large-scale study is required.

Therefore, we hypothesized that older adults with higher oral function would participate in social activities more frequently and aimed to examine the relationship between oral function and social participation among older adults living in the community.

## 2. Materials and Methods

### 2.1. Participants

In Japan, all individuals aged 75 years and older are enrolled in the long-life medical care system insurance and can undergo a free annual oral health checkup. In this study, the data used were obtained from the Shimane Extended Union of Medical Care System for Latter-Stage Elderly People, and data related to oral health checkups were processed to ensure anonymity. For this cross-sectional study, the participants were aged 75 years and older, residing in Shimane Prefecture, Japan, and had at least one oral health checkup between 1 April 2020 and 31 March 2022. The exclusion criteria were the following: 1. those who did not respond to the health assessment questionnaire for older adults and 2. those with missing values in the dental and oral health examination data. Within the two-year period, 55,528 participants underwent a general health checkup (Shimane Extended Union of Medical Care System for Latter-Stage Elderly People), and 14,431 participants underwent an oral health checkup. Among them, 6095 underwent both types of checkups during the study period and were thus included as initial participants. After data cleansing, including the exclusion of participants who had participated in the checkups multiple times and those with missing data, the final study population was reduced to 4196.

### 2.2. Data Collection

#### 2.2.1. Background Data

We collected data on age (years), sex (male/female), body mass index (BMI), and lower leg circumference (cm).

#### 2.2.2. Data on the Oral Cavity

We collected the following oral information: number of remaining teeth, periodontal tissue status, masticatory function, tongue range of motion, articulation, swallowing function, oral hygiene status, dry mouth, and daily frequency of tooth brushing. All the oral examinations were conducted by dentists and dental hygienists.

##### Number of Remaining Teeth

The remaining teeth were determined by visual examination and palpation.

##### Periodontal Tissue Status

The condition of periodontal tissues was determined by a dentist [20,21]. The dentists assessed tooth mobility (four levels: M0–M3) via palpation. Moreover, after visual inspection for redness, swelling, bleeding, suppuration of periodontal tissues, and calculus deposition, the overall condition of the site with the most advanced periodontal disease in the oral cavity was determined on a scale of normal or mild, moderate, severe, and no target teeth (edentulous jaws). The judgments were made regardless of the number of teeth in the edentulous jaw.

##### Masticatory Function

Masticatory function was evaluated using “Sugarless Fine Gummy jellies” (Fine Co., Ltd., Tokyo, Japan). One gummy was chewed for 15 s, and the number of crushed pieces of 3 mm or larger was counted to obtain the gummy 15 s value.

##### Tongue Mobility

Tongue mobility was considered poor if, when instructed by the dentist or dental hygienist to move the tongue forward, backward, and side to side, the patient was unable to extend the tongue beyond the lips.

##### Articulation

The dentist or dental hygienist asked the patient to read the sentence “panda-no-takara-mono” and conducted a monosyllabic listening test of “pa,” “ta,” “ka,” and “ra.” The patient was judged as deficient if there was one indistinct syllable.

##### Swallowing Function

Swallowing function was evaluated by recording the total time taken to complete three consecutive dry swallows, following a sip of water taken just before the test began (integrated time for three consecutive swallows).

##### Oral Hygiene Status

Oral hygiene status was evaluated on a four-point scale (good, needs attention, not so good, and not good) based on the overall condition of dental plaque adhesion, tongue coating, breath odor, denture cleaning, and cleanliness. Dental plaque adhesion was classified into four levels: “clean,” “moderate,” “abundant,” and “no remaining teeth,” and tongue coating was classified into three levels: “clean,” “moderate,” and “abundant.” Breath odor was classified into three levels: “not noticeable,” “mild,” and “strong.” Denture cleaning and cleanliness were classified into four levels: “clean,” “moderate,” “dirty,” and “no dentures.” If one or more of the ranks were dirtiest in each category, it was judged to be defective.

##### Dry Mouth

The method for determining dry mouth was based on one of the following three criteria: (1) If two or more of the following items were answered in the questionnaire: “mouth feels dry,” “tongue hurts,” and “taste has decreased.” (2) If they took more than 5 medications per day or had poor nutritional status and answered at least 1 of the following: “mouth feels dry,” “tongue hurts,” or “taste has decreased.” (3) If the patient responded “mouth feels dry” in the medical questionnaire and suspected xerostomia by visual and palpatory examination by a dentist, the patient was classified as dry mouth.

##### Daily Frequency of Tooth Brushing

The number of times patients brushed their teeth and cleaned their dentures daily was surveyed in the questionnaire.

#### 2.2.3. Questionnaire

Health assessment questionnaires for older adults, which have been used by local governments throughout Japan since April 2020, were used for the interviews. The social participation items in the questionnaire were, “do you go out at least once a week?” and “do you regularly meet with family or friends?”. “Going out” has been validated in a previous qualitative study as a question related to the social participation of the older adults [22]. Additionally, “meeting with family or friends” is a survey item used in studies examining the correlation with independence among older adults [23].

### 2.3. Statistical Analysis

Descriptive statistics were calculated as medians and percentages according to variables. Groups were divided into two groups: (1) those who went out at least once a week and those who did not go out at least once a week, (2) those who regularly met with family or friends and those who did not regularly meet with family or friends. Univariate and multivariate analyses were performed using logistic regression. Multicollinearity was assessed using the variance inflation factor (VIF), with a cutoff of 10. When multicollinearity was detected, one of the correlated variables was selected for inclusion in the model. Based on the previous literature, factors strongly suggested to be associated with oral disease were identified and used to select explanatory variables for multivariate analysis [24,25]. The objective variable was social participation, and the explanatory variables were age, sex, lower leg circumference, masticatory function, swallowing function, oral hygiene status, periodontal tissue status, dry mouth, and daily frequency of tooth brushing. Statistical analyses were performed using SPSS (version 27; SPSS Japan Inc., Tokyo, Japan). Statistical significance was set at less than 0.05.

## 3. Results

### 3.1. Participant Characteristics

The analysis included 4196 cases, excluding 513 cases with missing data, from a total of 4709 medical checkups (Table 1). The study included 1848 males and 2348 females. The median age was 78.0 (25–75 percentile: 77.0–81.0) years. The median BMI was 22.5 (25–75 percentile: 20.5–24.5) kg/m^2^. The median lower leg circumference was 33.0 (25–75 percentile: 31.0–35.0) cm. The median number of remaining teeth was 21.0 (25–75 percentile:12.0–26.0). The periodontal tissue status was as follows: 855 were healthy, 1729 had mild periodontal disease, 1216 had moderate periodontal disease, 158 had severe periodontal disease, and 238 were edentulous. The median masticatory function was 15.0 (25–75 percentile: 5.0–25.0). Dry mouth was absent in 3796 participants and present in 400. Tongue mobility was good in 4182 participants (99.7%) and poor in 14 participants (0.3%). The articulation Pa was good in 4188 (99.8) participants and unclear in 8 participants (0.2%). Articulation Ta was good in 4179 (99.6%) participants and unclear in 17 participants (0.4%). The articulation Ka was good in 4185 (99.7%) participants and unclear in 11 (0.3%) participants. Articulation Ra was good in 4174 (99.5%) participants and unclear in 22 (0.5%) participants. The median swallowing function was 13.0 (25–75 percentile: 10.0–19.0) s. The oral hygiene status was good in 2293 participants (54.6%), 1608 (38.3%) needed attention, 252 (6.0%) were not as good, and 43 (1.0%) were poor. The results of the questionnaire are shown in Table 2.

### 3.2. Comparison of Each Variable in Groups with and Without Social Participation

Significant differences were found between the groups that went out at least once a week and those that did not go out at least once a week, age, number of remaining teeth, masticatory function, periodontal tissue status, swallowing function, lower leg circumference, oral hygiene status, and dry mouth (*p* < 0.05, Table 3). Significant differences in sex, age, BMI, masticatory function, periodontal health, lower leg circumference, and oral hygiene status were observed between the groups that did and did not have regular meetings with family or friends (*p* < 0.05, Table 4).

### 3.3. Relationship Between Oral Function and Social Participation

Multivariate analysis of the presence or absence of going out at least once a week revealed significant correlation in age (OR = 0.954; 95%CI = 0.915–0.994; *p*-value = 0.026), lower leg circumference (OR = 1.052; 95%CI = 1.007–1.098; *p*-value = 0.022), masticatory function (OR = 1.021; 95%CI = 1.009–1.033; *p*-value < 0.001), and oral hygiene status (OR = 0.828; 95%CI = 0.696–0.985; *p*-value = 0.033, Table 5). Furthermore, significant correlations were found in sex (OR = 2.100; 95%CI = 1.549–2.848; *p*-value < 0.001), lower leg circumference (OR = 1.079; 95%CI = 1.026–1.135; *p*-value = 0.003), masticatory function (OR = 1.021; 95%CI = 1.007–1.034; *p*-value = 0.002), swallowing function (OR = 0.988; 95%CI = 0.976–0.999; *p*-value = 0.036), and oral hygiene status (OR = 0.678; 95%CI = 0.559–0.822; *p*-value < 0.001) with respect to whether or not they regularly meet with family or friends (*p* < 0.05, Table 6).

## 4. Discussion

The main finding of this study is the relationship between masticatory function and social participation. Masticatory function refers to the oral ability to break down food into a swallowable form, involving coordinated movements not only of the teeth but also of the entire oral cavity. It has been reported that factors that contribute to the decline of masticatory function are related to loss of teeth and muscle weakness, such as frailty and sarcopenia [26]. A study in England reported that the complete loss of remaining teeth in older adults was associated with a solitary living environment [27]. It has also been reported that those who participate in social activities have a 1.3-fold more remaining teeth than those who do not [28]. Moreover, poor oral function is reportedly associated with worse social withdrawal among older adults [29]. The study results are consistent with those of other studies, as objective masticatory ability has also been reported to be associated with higher daily living ability [30]. Although this study was cross-sectional and therefore cannot establish causality, previous research suggests that the observed phenomenon may be explained by a decline in masticatory function leading to reduced cognitive function and motivation, which in turn impacts social participation [31]. A previous study investigating a population similar to that in this study reported that the number of teeth and masticatory function decline with age and that reduced masticatory function is associated with a lower frequency of eating out [32,33]. Given that this study involved a group of relatively healthy older adults, a decline in masticatory function may also influence cognitive function, motivation, and social participation.

A minor finding of this study is the association between oral hygiene status and social participation. Dental caries and periodontal disease are the leading causes of tooth loss worldwide [34]. Oral hygiene status has been reported to be associated with cognitive decline among community-dwelling older adults requiring long-term care [35]. An association has also been demonstrated between social isolation and oral hygiene among older adults living independently [25]. Oral hygiene status was associated with social activity; however, one possible cause could be the association of halitosis with social activity. People who experience a psychological burden of halitosis have been reported to fear social opportunities and inhibit social activities [24]. Poor oral hygiene due to dental caries and periodontal disease is remarkably associated with eating alone, and a small number of teeth has been reported to reduce the variety of foods available, resulting in a lower frequency of eating out [33,36]. Hence, it seems reasonable to conclude that poor oral hygiene is associated with social activity. Hence, an association between oral hygiene status and social participation is reasonable in this study as well. However, mental health conditions such as depression may serve as an intermediate factor associated with both oral hygiene status and social participation [37]. Additionally, as oral literacy and poor oral hygiene have been noted in those with low social participation, it seems possible that oral hygiene status, oral function, and social participation may decline together while mutually influencing each other [38]. This study also suggested that older males over 75 years and older may be less likely to participate in society due to poor oral function [39]. In particular, older males, especially those living alone in rural regions, have multiple risk factors and need to be aware of the decline in oral function.

In this study, two questionnaire items, “Do you go out at least once a week?” and “Do you usually meet with family or friends?” were employed as indicators of social participation. In Japan, these two questions were used as indicators of social participation and, for example, as outcomes in social intervention studies [40]. However, these two questions have not been validated as questionnaires, and whether they are appropriate indicators of social participation remains unclear. In the fields of medical research, rehabilitation, and public health, tools such as the reintegration to normal living index and the community integration questionnaire have been developed to assess “social participation” as an outcome, and their validity and reliability are well established [41,42]. To the best of our knowledge, no validated measurement tool currently exists that directly incorporates the concept of “social participation” into the assessment of oral diseases. Moreover, the possibility that social participation may vary according to the cultural heterogeneity within a country or region should be considered. Intervention studies on social isolation in Spain have employed going out and contacting peers, including family and friends, as outcomes [43]. Meeting with friends was used to define social participation in a study on people with activity limitations in the Netherlands [23]. The above literature review suggests that “going out” and “meeting people” are less affected by cultural heterogeneity. Other social participation outcomes may also include participation in community clubs and volunteer activities. As club activities are strongly influenced by local characteristics and the communities in which they live, different research results may be obtained. Therefore, this study suggests that it is necessary to develop “an indicator to evaluate social activities involving oral function.”

Community-dwelling older adults are the target population for this study. The target population for this study included a greater number of females than males, and being female was associated with social activity. The study results are consistent with previous reports, as females were reported to be more socially active in previous reports [44]. The cutoff values for sarcopenia screening for lower leg circumference are <34 cm for males and 33 cm for females [45], and many subjects were already suspected of having sarcopenia. The average number of remaining teeth was 18.1 on average for those aged 75–79 years according to the Survey of Actual Conditions of Dental Diseases (2022), and many older adults had more than 20 remaining teeth and were able to chew adequately. Regarding periodontal disease, 56.0% of the older adults had periodontal pockets of 4 mm or larger, according to the Survey of Dental Diseases (2022); however, approximately 60% of the subjects had healthy or mild periodontal disease, indicating that many of them had better periodontal conditions than average. The cutoff value for masticatory function was set at 12 in previous studies, and the median value was 15, which was higher than 12, suggesting that many participants had good masticatory function. Swallowing function was measured as the time required to perform three empty swallows, with a median of 13 s in this study. The repetitive salivary swallowing test was considered normal at three or more swallows in 30 s, and the target population was considered capable of swallowing three or more times in 30 s. Altogether, these results suggest that the subject population in this study was a group of healthy late-aged individuals who could undergo health checkups. Therefore, while the generalization of the results of this study can be employed to healthy older adults living in the community in Japan, the adaptation of the study results should be carefully considered because cultural community formation in social participation differs internationally from country to country.

This study has three limitations. First, the target population was relatively healthy older adults who were able to receive community health checkups. In this study, being certified to require long-term nursing care under Japanese National Health Insurance was not used as an exclusion criterion. Such participants were included. Although cognitive function was not an exclusion criterion, participants were required to complete the questionnaire independently. Therefore, participants with apparent cognitive impairment were excluded. Participants who were hospitalized for extended periods or resided in long-term care facilities were excluded because they were not eligible for health checkups. This limitation represents a form of bias known as the healthy volunteer bias, which is challenging to control using statistical analyses. Consequently, the findings of this study can be generalized to other regions of Japan with similar aging populations. Second, this was a cross-sectional study, and causal relationships could not be verified. Third, oral function examination is a unique method of oral health checkups in Shimane Prefecture, Japan. The recommended method for evaluating oral function is the measurement of a diagnostic item for oral hypofunction, which was included in the National Health Insurance database in 2018. However, we used data from group health checkups conducted by the Shimane Dental Association in 2015. Although this is a simple and comprehensive examination of oral function, and cutoff values have been established based on studies of community-based data, the reliability and validity of the respective data remain questionable. Although we explored the data cross-sectionally in this study, future studies should examine the data longitudinally to verify the causal relationships.

## 5. Conclusions

The results of this study suggest that the masticatory, oral hygiene, and swallowing functions are associated with social participation. It is considered necessary for oral health care providers to develop oral health care activities while providing opportunities for social participation of the older adults, such as comprehensive community care, to maintain and promote oral health status and function.

## Figures and Tables

**Table 1:** Demographic characteristics (n = 4196).

Variables	Categories	n (%)	Median [25–75 Percentile]
Age (years)			78.0 [77.0–81.0]
Sex	Male	1848 (44.0)	
	Female	2348 (56.0)	
Body mass index (kg/m^2^)			22.5 [20.5–24.5]
Number of remaining teeth			21.0 [12.0–26.0]
Masticatory function (gummy 15 s)			15.0 [5.0–25.0]
Periodontal tissue status	Healthy	855 (20.4)	
	Mild periodontal disease	1729 (41.2)	
	Moderate periodontal disease	1216 (29.0)	
	Severe periodontal disease	158 (3.8)	
	Edentulism	238 (5.7)	
Swallowing function			13.0 [10.0–19.0]
Lower leg circumference (cm)			33.0 [31.0–35.0]
Oral hygiene status	Good	2293 (54.6)	
	Caution required	1608 (38.3)	
	Not very good	252 (6.0)	
	Very dirty	43 (1.0)	
Dry mouth	No problem	3796 (90.5)	
	Drying	400 (9.5)	
Tongue mobility	Good	4182 (99.7)	
	Bad	14 (0.3)	
Articulation Pa	Good	4188 (99.8)	
	Unclear	8 (0.2)	
Articulation Ta	Good	4179 (99.6)	
	Unclear	17 (0.4)	
Articulation Ka	Good	4185 (99.7)	
	Unclear	11 (0.3)	
Articulation Ra	Good	4174 (99.5)	
	Unclear	22 (0.5)	
Daily frequency of tooth brushing	Once	799 (19.0)	
	Twice	1809 (43.1)	
	3 times or more	1546 (36.8)	
	Do not brush teeth	42 (1.0)	

**Table 2:** Health assessment questionnaire for older adults (n = 4196).

Variables	Categories	n (%)
(1) How is your current health condition? (Health status)	Good	1073 (25.6)
	Somewhat good	786 (18.7)
	Normal	1923 (45.8)
	Not very good	382 (9.1)
	Not good	32 (0.8)
(2) Are you satisfied with your daily life? (Mental health status)	Satisfied	2001 (47.7)
	Somewhat satisfied	1846 (44.0)
	Somewhat dissatisfied	319 (7.6)
	Dissatisfied	30 (0.7)
(3) Do you regularly eat three meals a day? (Eating habits)	Yes	4098 (97.7)
	No	98 (2.3)
(4) Compared to 6 months ago, do you find it more difficult to eat tough or solid foods? (Oral function)	Yes	941 (22.4)
	No	3255 (77.6)
(5) Do you find yourself choking on tea or soup? (Oral function)	Yes	871 (20.8)
	No	3325 (79.2)
(6) Have you lost 2–3 kg or more in the past 6 months? (Weight changes)	Yes	52 (12.4)
	No	3674 (87.6)
(7) Do you think your walking speed has slowed down as compared to before? (Exercise/Fall)	Yes	2321 (55.3)
	No	1875 (44.7)
(8) Have you fallen down previously in the past year? (Exercise/Fall)	Yes	774 (18.4)
	No	3422 (81.6)
(9) Do you exercise (take walks, etc.) at least once a week? (Exercise/Fall)	Yes	2326 (55.4)
	No	1870 (44.6)
(10) Do people around you comment on your forgetfulness, e.g., say to you, “You are always asking the same thing.” (Cognitive function)	Yes	589 (14.0)
	No	3607 (86.0)
(11) There are times when you do not remember today’s date. (Cognitive function)	Yes	959 (22.9)
	No	3237 (77.1)
(12) Do you smoke? (Smoking)	Smoking	138 (3.3)
	Not smoking	3334 (79.5)
	Smoked in the past	724 (17.3)
(13) Do you go out at least once a week? (Social participation)	Yes	3914 (93.3)
	No	282 (6.7)
(14) Do you regularly meet with family or friends? (Social participation)	Yes	3986 (95.0)
	No	210 (5.0)
(15) When you are not feeling well, do you have someone close by to talk to? (Social support)	Yes	3943 (94.0)
	No	257 (6.0)

**Table 3:** Comparison between group of going out at least once a week or not.

Variables		N (%) or Median [25–75 Percentile]		*p*-Value
		Yes (n = 3914)	No (n = 282)	
Age (years)		78.0 [76.0–81.0]	79.0 [77.0–82.0]	<0.01 *
Sex	Male	1733 (44.3)	115 (40.8)	0.25
	Female	2181 (55.7)	167 (59.2)	
Body mass index (kg/m^2^)		22.5 [20.6–24.5]	22.3 [20.1–24.5]	0.23
Number of remaining teeth		21.0 [12.0–26.0]	18.0 [7.0–24.0]	<0.01 *
Masticatory function (gummy 15 s)		15.0 [6.0–25.0]	9.5 [2.0–18.0]	<0.01 *
Periodontal tissue status		2.0 [2.0–3.0]	2.0 [2.0–3.0]	<0.01 *
Swallowing function		13.0 [10.0–19.0]	14.0 [10.0–20.0]	0.05 *
Lower leg circumference (cm)		33.0 [31.2–35.0]	33.0 [31.0–35.0]	<0.01 *
Oral hygiene status	Good	2150 (54.9)	143 (50.7)	<0.01 *
	Caution requires	1505 (38.5)	103 (36.5)	
	Not very good	228 (5.8)	24 (8.5)	
	Very dirty	31 (0.8)	12 (4.3)	
Dry mouth	No problem	3554 (90.8)	242 (85.8)	<0.01 *
	Drying	360 (9.2)	40 (14.2)	
Tongue mobility	Good	3902 (99.7)	280 (99.3)	0.26
	Bad	12 (0.3)	2 (0.7)	
Articulation Pa	Good	3907 (99.8)	281 (99.6)	0.51
	Unclear	7 (0.2)	1 (0.4)	
Articulation Ta	Good	3899 (99.6)	280 (99.3)	0.41
	Unclear	15 (0.4)	2 (0.7)	
Articulation Ka	Good	3903 (99.7)	282 (100)	0.37
	Unclear	11 (0.3)	0 (0)	
Articulation Ta	Good	3894 (99.5)	280 (99.3)	0.66
	Unclear	20 (0.5)	2 (0.7)	
Daily frequency of tooth brushing	Once	738 (18.9)	61 (21.6)	0.02 *
	Twice	1638 (1683)	126 (44.7)	
	3 times or more	1458 (1458)	88 (31.2)	
	Do not brush teeth	35 (35)	7 (2.5)	

* *p* < 0.05.

**Table 4:** Comparison between group of regularly meeting family or friends or not.

Variables		N (%) or Median [25–75 Percentile]		*p*-Value
		Yes (n = 3986)	No (n = 210)	
Age (years)		78.0 [76.0–81.0]	79.0 [77.0–82.0]	0.03 *
Sex	Male	1728 (43.4)	120 (57.1)	<0.01 *
	Female	2258 (56.6)	90 (42.9)	
Body mass index (kg/m^2^)		22.5 [20.6–24.5]	22.1 [20.0–24.0]	0.04 *
Number of remaining teeth		21.0 [12.0–26.0]	19.0 [8.6–25.2]	0.12
Masticatory function (gummy 15 s)		15.0 [6.0–25.0]	10.5 [3.0–21.0]	<0.01 *
Periodontal tissue status		2.0 [2.0–3.0]	2.0 [2.0–3.0]	<0.01 *
Swallowing function		13.0 [10.0–19.0]	14.0 [9.0–20.0]	0.31
Lower leg circumference (cm)		33.0 [31.2–35.0]	32.6 [30.7–35.0]	<0.01 *
Oral hygiene status	Good	2210 (55.4)	83 (39.5)	<0.01 *
	Caution requires	1510 (37.9)	98 (46.7)	
	Not very good	229 (5.7)	23 (11.0)	
	Very dirty	37 (0.9)	6 (2.9)	
Dry mouth	No problem	3607 (90.5)	189 (90.0)	0.81
	Drying	379 (9.5)	21 (10.0)	
Tongue mobility	Good	3972 (99.6)	210 (100.0)	0.39
	Bad	14 (0.4)	0 (0)	
Articulation Pa	Good	3979 (99.8)	209 (99.5)	0.33
	Unclear	7 (0.2)	1 (0.5)	
Articulation Ta	Good	3969 (99.6)	210 (100.0)	0.34
	Unclear	17 (0.4)	0 (0)	
Articulation Ka	Good	3975 (99.7)	210 (100.0)	0.45
	Unclear	11 (0.3)	0 (0)	
Articulation Ra	Good	3965 (99.5)	209 (99.5)	0.92
	Unclear	21 (0.5)	1 (0.5)	
Daily frequency of tooth brushing	Once	741 (18.6)	58 (27.6)	<0.01 *
	Twice	1725 (43.3)	84 (40.0)	
	3 times or more	1483 (37.2)	63 (30.0)	
	Do not brush teeth	37 (0.9)	5 (2.4)	

* *p* < 0.05.

**Table 5:** Factors associated with going out at least once a week using logistic regression analysis.

Variables	Categories	Univariate		Multivariate	
		Odds (95% CI)	*p*-Value	Odds (95% CI)	*p*-Value
Age (years)		0.93 (0.89–0.97)	<0.01 *	0.95 (0.91–0.99)	0.03 *
Sex	Male	1 (ref)		1 (ref)	
	Female	0.87 (0.68–1.11)	0.25	1.04 (0.80–1.35)	0.77
Lower leg circumference (cm)		1.07 (1.03–1.12)	<0.01 *	1.05 (1.01–1.10)	0.02 *
Periodontal tissue status	Healthy	1 (ref)		1 (ref)	
	Mild periodontal disease	0.82 (0.57–1.18)	0.29	0.86 (0.59–1.25)	0.43
	Moderate periodontal disease	0.71 (0.49–1.04)	0.08	0.85 (0.56–1.27)	0.42
	Severe periodontal disease	0.34 (0.20–0.58)	<0.01 *	0.53 (0.29–0.98)	0.04 *
	Edentulism	0.51 (0.30–0.86)	0.01 *	0.76 (0.44–1.33)	0.34
Masticatory function (gummy 15 s)		1.03 (1.02–1.04)	<0.01 *	1.02 (1.01–1.03)	<0.01 *
Swallowing function		0.99 (0.98–1.00)	0.06	1.00 (0.99–1.01)	0.51
Oral hygiene status	Good	1 (ref)		1 (ref)	
	Caution required	0.97 (0.75–1.26)	0.83	1.08 (0.81–1.44)	0.60
	Not very good	0.63 (0.40–0.99)	0.05 *	0.82 (0.50–1.34)	0.42
	Very dirty	0.17 (0.09–0.34)	<0.01 *	0.29 (0.13–0.61)	<0.01 *
Dry mouth		0.61 (0.43–0.87)	<0.01 *	0.71 (0.50–1.03)	0.07

CI: confidence interval, * *p* < 0.05.

**Table 6:** Factors associated with regularly meeting family or friends using logistic regression analysis.

Variables	Categories	Univariate		Multivariate	
		Odds (95% CI)	*p*-Value	Odds (95% CI)	*p*-Value
Age (years)		0.95 (0.91–0.99)	0.03 *	0.97 (0.92–1.02)	0.19
Sex	Male	1 (ref)		1 (ref)	
	Female	1.74 (1.32–2.31)	<0.01 *	2.04 (1.50–2.78)	<0.01 *
Lower leg circumference (cm)		1.06 (1.01–1.11)	0.01 *	1.08 (1.03–1.14)	<0.01 *
Periodontal tissue status	Healthy	1 (ref)		1 (ref)	
	Mild periodontal disease	0.70 (0.45–1.07)	0.10	0.87 (0.56–1.37)	0.55
	Moderate periodontal disease	0.53 (0.34–0.82)	<0.01 *	0.82 (0.51–1.33)	0.43
	Severe periodontal disease	0.43 (0.21–0.86)	0.02 *	0.85 (0.39–1.84)	0.70
	Edentulism	0.80 (0.38–1.67)	0.55	1.51 (0.69–3.27)	0.30
Masticatory function (gummy 15 s)		1.02 (1.01–1.04)	<0.01 *	1.02 (1.01–1.03)	<0.01 *
Swallowing function		0.99 (0.97–0.99)	<0.01 *	0.99 (0.98–0.99)	0.03 *
Oral hygiene status	Good	1 (ref)		1 (ref)	
	Caution required	0.58 (0.43–0.78)	<0.01 *	0.69 (0.50–0.96)	0.03 *
	Not very good	0.37 (0.23–0.61)	<0.01 *	0.56 (0.33–0.96)	0.03 *
	Very dirty	0.23 (0.10–0.56)	<0.01 *	0.40 (0.15–1.04)	0.06
Daily frequency of tooth brushing	Once	1 (ref)		1 (ref)	
	Twice	1.61 (1.14–2.27)	<0.01 *	1.28 (0.89–1.84)	0.18
	3 times or more	1.84 (1.28–2.66)	<0.01 *	1.42 (0.96–2.09)	0.08
	Do not brush teeth	0.58 (0.22–1.53)	0.27	0.56 (0.20–1.52)	0.25

CI: confidence interval, * *p* < 0.05.

## Data Availability

The data supporting the findings of this study are not publicly available because the study protocol did not include a provision for publicly shared data. Approval must be obtained from the Medical Research Ethics Committee of Shimane University Faculty of Medicine (kenkyu@med.shimane-u.ac.jp) to request the provision of de-identified data. Moreover, individual permission was obtained from the Shimane Extended Union of Medical Care System for Latter-Stage Elderly People, who managed the original data for this study.
